# Job preferences of undergraduate nursing students in eastern China: a discrete choice experiment

**DOI:** 10.1186/s12960-018-0335-3

**Published:** 2019-01-03

**Authors:** Tongtong Liu, Shunping Li, Renyong Yang, Shimeng Liu, Gang Chen

**Affiliations:** 1 The Affiliated Hospital of Qingdao University, Qingdao, 266000 China; 2 School of Health Care Management, Shandong University, Jinan, 250012 China; 3 NHC Key Laboratory of Health Economics and Policy Research (Shandong University), Jinan, 250012 China; 4 Jining Medical University, Jining, 272067 China; 5 College of Medicine and Public Health, Flinders University, Adelaide, 5042 Australia

**Keywords:** Discrete choice experiments, Job preferences, Undergraduate nursing students, China

## Abstract

**Background:**

Shortage and mal-distribution of nursing human resources is an intractable problem in China. There is an urgent need to explore the job preferences of undergraduate nursing students. The main aim of this study is to investigate the stated preferences of nursing students when choosing a job.

**Methods:**

A discrete choice experiment (DCE) was conducted to assess job preferences of the final year undergraduate nursing students from four medical universities/colleges in Shandong Province, China. Job attributes include location, monthly income, *bianzhi* (which refers to the established posts and can be loosely regarded as state administrative staffing), career development and training opportunity, work environment and working strength. Mixed logit models were used to analyze the DCE data.

**Results:**

A total of 445 undergraduate nursing students were included in the main DCE analysis. They demonstrated higher preference for a job with higher monthly income, and the probability of choosing a rural job would increase to 92.8% if monthly income increased from RMB 2000 (US$ 296) to RMB 8000 (US$ 1183). They expressed higher stated preferences for a job which required light working strength and with excellent work environment over other non-economic attributes. Among all attributes, location was the least important attribute. Subgroup analysis showed that students who came from city or county and whose family income was more than RMB 50 000 (US$ 7396) were significantly willing to pay more monthly income for a job in city.

**Conclusions:**

This study confirmed that economic and non-economic factors both affected the job choices of the students. These results may be more effective for policymakers to perfect the employment policies and design strategies to attract more nursing students taking jobs in rural areas.

**Electronic supplementary material:**

The online version of this article (10.1186/s12960-018-0335-3) contains supplementary material, which is available to authorized users.

## Background

In 2006, the World Health Organization issued the World Health Report, with the theme of “working together for health,” to indicate the negative impact of human resources shortages in global health care [1]. Currently, the scarcity of skilled nurses is being highlighted as one of the biggest obstacles to achieving health system effectiveness both in developed and developing countries [2–4]. China also faces the severe challenges of nursing shortages [3]. In 2013, there was 2.05 nurses per 1000 population, which was much lower than the world average of 2.86 [5]. It was estimated that there were 3.51 million registered nurses in China in 2016, and the ratio of doctors to nurses was only 1:1.10 [6], lower than the targeting ratio of 1:1.25, which is documented in the Outline of National Medical and Health Service System (2015–2020).

Mal-distribution of nursing human resources and other healthcare providers is also a challenging issue in both developed and developing countries [7, 8]. Globally, 50% of the population live in rural areas and served by 38% of the total nursing workforce [9]. Similarly, in China, the density of nurses in urban areas is much higher than in rural areas, and the gap between urban and rural areas is enlarging. From 2010 to 2015, registered nurses per 1000 population have increased from 3.09 to 4.75 in urban areas and only increased from 0.89 to 1.50 in rural areas [6]. It should also be noted that within the hierarchical medical system, the number of registered nurses in basic medical and health institutions (including community health service centers in urban areas, and township hospitals and village clinics in rural areas) is much lower than the secondary and tertiary hospitals (0.70 million versus 2.81 million) [6]. In addition, the job transfer rate of Chinese nurses in basic medical and health institutions is high, and some of them move to other positions, such as administration and logistics [10].

Nursing staff is an important guarantee for improving healthy China and providing quality, efficient, and convenient health services. The shortage and mal-distribution of nursing staff are partly responsible for the increasing workload of existing nurses, which may decrease the quality of health services and patient’s satisfaction, even cause conflict between nurses and patients [11].

Both existing nurses and final year nursing students who will soon be on the job market are important human resources for health in the long term. There has been a greater demand from health institutions for nurses, especially for nurses with higher educational levels [12]. Although medical universities/colleges have expanded enrollment levels to meet their demands, the shortage of nurses is still a severe problem in China [12]. This might be explained by the fact that the majority of medical universities/colleges graduates compete for tertiary hospitals, in which their salaries, career development, and work environment are superior to those offered by basic medical and health institutions [13]. How to attract and retain undergraduate nursing students in basic medical and health institutions especially in rural areas is an extremely important problem for policymakers.

Discrete choice experiment (DCE) is a quantitative approach that combines random utility theory [14], consumer theory [15], experimental design theory [16], and econometric analysis [17]. DCEs have been more widely applied to health care areas, primarily to value patient experiences or assess trade-offs between health outcomes and patient experiences [18]. In recent years, the number of studies that used DCEs to explore the job preferences of health care providers, including nurses and nursing students, has been increasing [7, 19–27], and most of them are set in developing countries. All studies demonstrate the influence of economic and non-economic factors for the job choices of the students. However, different studies include different job attributes, and the findings from these studies are unlikely to represent China’s special national conditions. So far, there is one DCE study which investigated the job preferences of nursing students in China; however, the study was based on 164 nursing students who took internship in a tertiary hospital [28]. This study showed that the aspects prioritized by nursing students when choosing a position in basic medical and health institutions were as follows in descending order: monthly income, children’s educational conditions, *bianzhi*, location, working conditions, career development, and training opportunity.

By using randomly selected nursing students, this study aims to identify what job characteristics affect job choices of final year undergraduate nursing students by using a DCE in eastern China. The findings of the study will provide valuable information for policymakers to design effective strategies to attract more nursing students taking jobs in rural areas, to consolidate, stabilize, enrich, and develop construction of nursing teams.

## Methods

### Setting and sample

This study was conducted in Shandong Province, which is located in eastern China, with a population of about 100 million [29]. In 2016, the gross regional product of Shandong Province amounted to RMB 6802 billion (US$ 1024 billion), ranking it as the third largest economy within China [29]. However, there are only 0.27 million registered nurses in Shandong Province, and the ratio of doctors and nurses is 1:1.10 which is ranked the 16th in China [6].

There are eight medical universities/colleges in Shandong Province [30], and five are provincial independent medical universities/colleges. Considering the different education system, we excluded Shandong University of Traditional Chinese Medicine. Four medical universities/colleges, namely Weifang Medical University, Binzhou Medical University, Jining Medical University, and Taishan Medical College were included. The final year undergraduate nursing students were selected in this study because they were considering various employment choices but had not yet made final decisions [31]. A cluster random sampling method was used, and we aimed to recruit a minimum of 100 respondents from each medical university/college [32]. Accordingly, two or three classes in each university/medical college were randomly selected depending on the number of final year undergraduate nursing students in each class.

### Discrete choice experiment design

Establishing the job attributes and corresponding levels is the first and key step in DCEs and initial identification based on literature reviews. Location, monthly income, training opportunity, promotion opportunities, *bianzhi* (which refers to the established posts and can be loosely regarded as state administrative staffing) [33], facility quality, workload, management style, and organizational culture have been commonly adopted and suitable for the Chinese health care system [7, 21–23, 26, 28, 31, 34–36]. Then, in-depth interviews were conducted with five professional nurses from different hospital levels. As a result, the condition of working night shift was added as the potential attributes. Two focus groups (seven participants per group) were then conducted among the final year undergraduate nursing students from Weifang Medical University. They were asked to discuss which attributes were considered the relative important factors in their job selection until they reached a consensus for the final attributes and their levels. In this process, we used the new attribute of a job which required light working strength to replace both the workload and the condition of working night shift. Meanwhile, facility quality, management style, and organizational culture were generalized into the attribute of work environment. Based on literature reviews, in-depth interview, focus group discussions, and the final review with two specialists on DCEs, six attributes and their levels were finally included within the DCE (Table 1). The final six attributes include a monetary attribute (monthly income) and five non-monetary attributes: location, *bianzhi*, career development and training opportunity, work environment, and work strength.

**Table 1:** Discrete choice experiment attributes and levels for final year undergraduate nursing students

Attributes	Definition	Levels
Location	Location means to work in different level hospitals of the different regions.	Township or village
		County
		City
Monthly income	Monthly income includes salary, bonus, and welfare benefits (pension scheme, basic medical insurance, unemployment insurance, employment injury insurance, maternity insurance, and housing accumulation funds).	RMB 2000
		RMB 5000
		RMB 8000
*Bianzhi*	*Bianzhi* means that the establishment of medical institutions, the number of staff, and distribution of duties which are allocated by financial departments are prepared by *bianzhi* departments at different levels.	No
		Yes
Career development and training opportunity	Career development and training opportunity includes the opportunities of promotion and participating in various professional training.	Insufficient
		Some
		Sufficient
Work environment	Work environment includes infrastructures, medical facilities and medicines of hospital, management style and cultural development of hospital, amenities (such as regular bus, canteen, and lounge), superior-subordinate relationship and colleagueship.	Poor: insufficient essential equipment, limited support by managers, poor superior-subordinate relationship and colleagueship.
		Normal: nearly sufficient essential equipment, partly support by managers, moderate superior-subordinate relationship and colleagueship.
		Excellent: sufficient essential equipment, full support by managers, harmonious superior-subordinate relationship and colleagueship.
Working strength	Working strength includes the workload in the daytime (whether they have enough time to complete duties) and the conditions of working overtime or night shift.	Heavy: barely enough time to complete duties, working overtime or night shift four times a week at least.
		Medium: nearly enough time to complete duties, working overtime or night shift twice or three times a week.
		Light: enough time to complete duties, working overtime or night shift once a week at most.

Note: According to the Organisation for Economic Co-operation and Development (OECD) data (https://data.oecd.org/conversion/exchange-rates.htm), the average annual exchange rate between US$ and RMB in 2017 was US$ 1 = RMB 6.76

A full factorial design will produce 3^5^ × 2 = 486 hypothetical job scenarios. A total of (486 × 485)/2 choice sets will be generated, and those are not feasible for a single individual to choose. Accordingly, 24 choice sets were constructed by using a D-efficient design (assuming zero priors for all attributes) with Ngene DCE design software [37]. In order to lessen the burden of participants, Ngene software was also employed to divide 24 choice sets into two versions. One of the choice sets in each version was included twice as a consistency test but the data of the repeated choice sets were not included in the final analysis. The two versions of questionnaires were randomly allocated to participants. An example of the choice sets was demonstrated in Table 2.

**Table 2:** An example of a discrete choice experiment question for assessing job preferences of the final year undergraduate nursing students

	Job A	Job B
Location	City	Township or village
Monthly income	RMB 8000	RMB 5000
*Bianzhi*	No	Yes
Career development and training opportunity	Some	Sufficient
Work environment	Excellent	Normal
Working strength	Medium	Light
Which job would you be more likely to choose?	☐	☐

### Data collection

A pilot investigation (*n* = 15) was carried out among final year undergraduate nursing students from Weifang Medical University in March 2017 to examine intelligibility, acceptability, and face validity of the questionnaire, and minor modifications were made to attribute definitions and levels. The formal investigation was conducted in the classroom setting between April and June 2017. The questionnaire consisted of two sections. The first section included general characteristics of participants. Furthermore, they were also asked to rank the job attributes from most important to least important. The second section contained 13 choice sets of hypothetical job scenarios. The DCE questionnaires were explained in detailed by one of the three trained researchers from Shandong University. Then, students finished the structured questionnaires by themselves. If they had any questions, researchers would help them. On average, participants took approximately 20 min to complete the survey.

The study has been approved by the Ethics Review Board of the School of Preventive Medicine, Shandong University (Reference No. 20170301). Informed consent was obtained from all participants prior to questionnaire administration.

### Data analysis

Responses were coded and double-entered into an electronic database using EpiData version 3.1 (EpiData Association, Odense, Denmark). Statistical analyses were performed using Stata version 13.1 (StataCorp LP, College Station, TX, USA). The general characteristics of the undergraduate nursing students were summarized as means and standard deviations or frequencies and percentages.

The theoretical basis for statistical analysis of DCE data is the random utility model. In this framework, it is assumed that individual *n* will opt for the option that is associated with the highest utility level. In this study, we analyzed the data using a mixed logit model which has the convenient properties of allowing for the preference heterogeneity and violation of the independence of irrelevant alternative assumption [18, 38]. All attributes were specified as having a random component. Monthly income was coded as a continuous variable, and other attributes were coded as dummy variables. Mixed logit models were used to analyze the variables using mixlogit command [39] and were specified with 500 Halton draws. The utility that individual *n* obtains from alternative *i* in choice set *t* is depicted as:

$$ {\mathrm{U}}_{\mathrm{nit}}=\left({\beta}_1+{\eta}_{1n}\right)\ \mathrm{monthly}\ \mathrm{income}+\left({\beta}_2+{\eta}_{2n}\right)\ \mathrm{location}\_\mathrm{county}+\left({\beta}_3+{\eta}_{3n}\right)\ \mathrm{location}\_\mathrm{city}+\left({\beta}_4+{\eta}_{4n}\right)\  bianzhi\_\mathrm{yes}+\left({\beta}_5+{\eta}_{5n}\right)\ \mathrm{opportunity}\_\mathrm{some}+\left({\beta}_6+{\eta}_{6n}\right)\ \mathrm{opportunity}\_\mathrm{sufficient}+\left({\beta}_7+{\eta}_{7n}\right)\ \mathrm{environment}\_\mathrm{normal}+\left({\beta}_8+{\eta}_{8n}\right)\ \mathrm{environment}\_\mathrm{excellent}+\left({\beta}_9+{\eta}_{9n}\right)\ \mathrm{strength}\_\mathrm{medium}+\left({\beta}_{10}+{\eta}_{10n}\right)\ \mathrm{strength}\_\mathrm{light}+{\varepsilon}_{\mathrm{nit}} $$

where *β*_*i*_ is a vector of coefficients representing the relative preference weights for each attribute level on average, *η*_*i*_ reflects the degree of heterogeneity among respondents, and *ε*_nit_ is unobservable random component.

Willingness to pay (WTP) represents a monetary measure of participants’ valuation for a certain attribute. WTP was the ratio of the coefficient between each attribute level and the monthly income attribute. The positive or negative WTP results indicated the portion of monthly income that participants would be willing to pay or to be compensated for an attribute level. We also conducted a simulation study to predict that the uptake rates of undergraduate nursing students for rural versus city jobs change as the levels of job attributes are changed (policy interventions).

## Results

A total of 554 final year undergraduate nursing students were recruited in the survey, 507 completed the majority of the questionnaire. The number of participants from four medical universities/colleges were 141 (Weifang), 107 (Binzhou), 139 (Jining), and 120 (Taishan), respectively. Participants who gave inconsistent answers to repeated choice sets were excluded from the main analysis (*n* = 62, 12.2%), and detailed results reported below were based on the remaining 445 undergraduate nursing students. Meanwhile, a sensitivity analysis including all 507 participants was conducted, and the results were not substantially significantly different (Additional file 1: Table S1).

### General characteristics

The general characteristics of respondents are presented in Table 3. There were no significant differences between participants who passed or failed the consistency test. The mean age of the analytical sample (*n* = 445) was 22.3 ± 1.2 years, and 410 (92.1%) students were between ages 21 and 24. Female (91.5%) were overwhelmingly predominant in the participants. Most of the students (69.4%) came from townships or villages, and 327 (73.5%) students had brothers or sisters. More than 90% of participants spent less than RMB 1500 per month, and approximately 60% participants indicated their annual family income as less than RMB 50 000. With regard to future plans, the majority of the students (*n* = 361) said they wanted to engage in nursing work after graduation.

**Table 3:** The general characteristics for final year undergraduate nursing students from four medical universities/colleges, n (%)

Characteristics	All (*n* = 507)	Participants who passed the consistency test (*n* = 445)	Participants who failed the consistency test (*n* = 62)	*χ* ^2^	*P* value
Gender					
Male	41 (8.1%)	38 (8.5%)	3 (4.8%)	1.003	0.317
Female	466 (91.9%)	407 (91.5%)	59 (95.2%)		
Hometown					
City	69 (13.6%)	61 (13.7%)	8 (12.9%)	3.400	0.183
County	80 (15.8%)	75 (16.9%)	5 (8.1%)		
Township or village	358 (70.6%)	309 (69.4%)	49 (79.0%)		
Only child					
Yes	132 (26.0%)	118 (26.5%)	14 (22.6%)	0.438	0.508
No	375 (74.0%)	327 (73.5%)	48 (77.4%)		
Consumption level (RMB per month)					
< 800	139 (27.4%)	120 (27.0%)	19 (30.7%)	1.573	0.455
800–1500	323 (63.7%)	283 (63.6%)	40 (64.5%)		
> 1500	45 (8.9%)	42 (9.4%)	3 (4.8%)		
Family income (RMB per year)					
< 30 000	157 (31.0%)	143 (32.1%)	14 (22.6%)	3.652	0.455
30 000–50 000	181 (35.7%)	154 (34.6%)	27 (43.5%)		
50 000–70 000	88 (17.3%)	76 (17.1%)	12 (19.4%)		
70 000–90 000	37 (7.3%)	34 (7.7%)	3 (4.8%)		
> 90 000	44 (8.7%)	38 (8.5%)	6 (9.7%)		
Your future plan after graduation					
Engage in nursing work	406 (80.1%)	361 (81.1%)	45 (72.6%)	2.588	0.274
Continue education	87 (17.1%)	72 (16.2%)	15 (24.2%)		
Others	14 (2.8%)	12 (2.7%)	2 (3.2%)		

Note: According to the OECD data, the average annual exchange rate between US$ and RMB in 2017 was US$ 1 = RMB 6.76

**Fig. 1:**
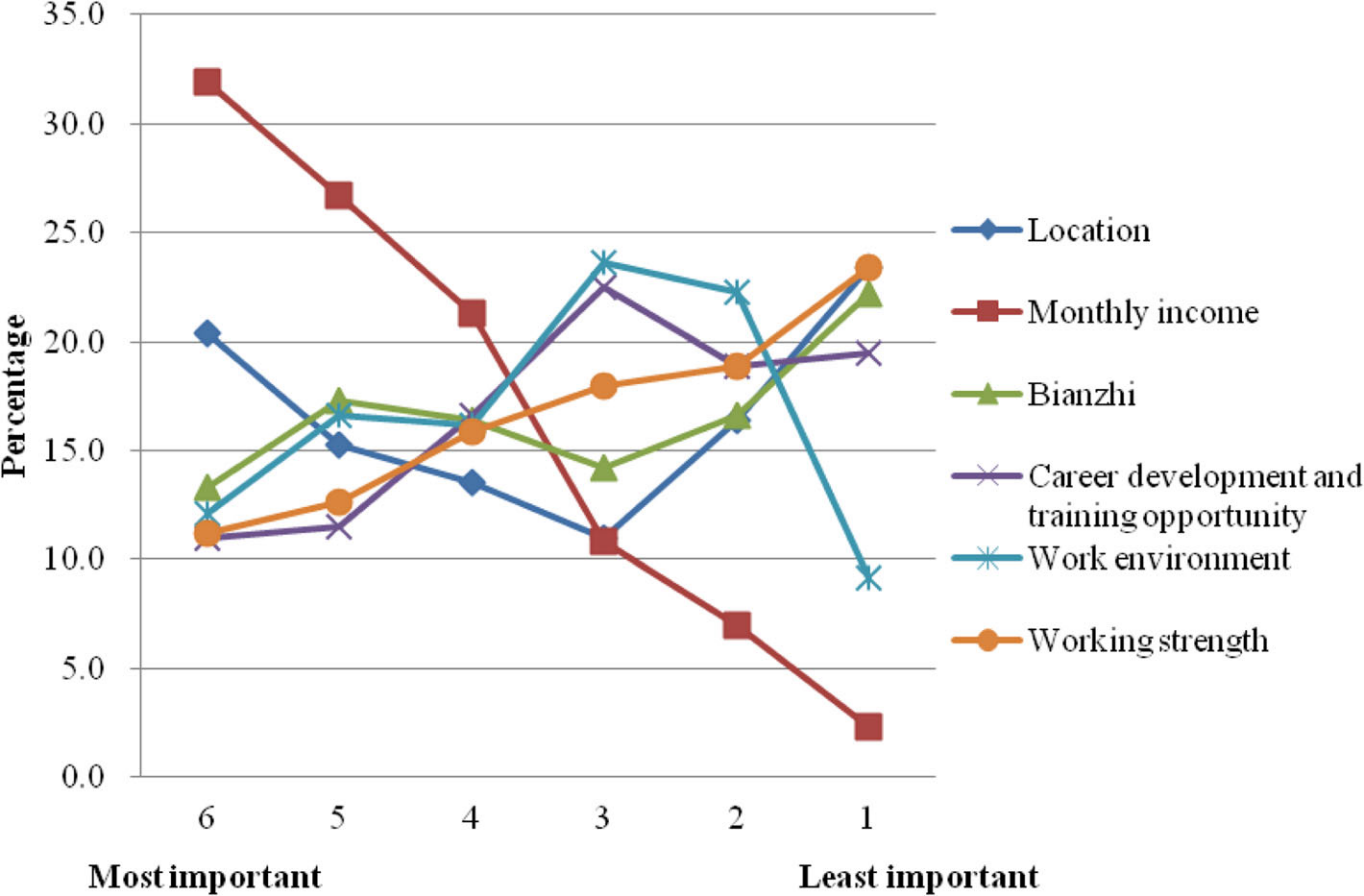
Figure that demonstrates the importance of job attributes

Results from the standalone ranking of importance of job attributes (which was asked prior to the DCE tasks) is showed in Fig. 1. On average, monthly income was regarded as the most important job attribute, followed by location.

### Job preferences among undergraduate nursing students

Table 4 shows the main results from the mixed logit model. The mean coefficients of all attributes were statistically significant and were of the expected sign. Among non-monetary attributes, the final year undergraduate nursing students expressed highest stated preferences for a job with light working strength (*β* = 1.013, *P* < 0.01), followed by an excellent work environment (*β* = 0.760, *P* < 0.01). Although a city location had a positive effect for the respondents (*β* = 0.339, *P* < 0.01), it did not appear to be as important as the other attributes.

**Table 4:** Results of a mixed logit model of DCE data from final year undergraduate nursing students

Attributes	Mean coefficient	Standard error	Standard deviation	Standard error
Monthly income	0.000483***	0.000022	0.000243***	0.000020
Location (ref: Township or village)				
County	0.292***	0.061	0.012	0.174
City	0.339***	0.067	0.499***	0.104
*Bianzhi* (ref: No)				
Yes	0.648***	0.059	0.635***	0.070
Career development and training opportunity (ref: Insufficient)				
Some	0.101*	0.060	0.055	0.179
Sufficient	0.652***	0.069	0.118	0.355
Work environment (ref: Poor)				
Normal	0.589***	0.064	0.008	0.146
Excellent	0.760***	0.064	0.021	0.156
Working strength (ref: Heavy)				
Medium	1.008***	0.067	0.025	0.154
Light	1.013***	0.072	0.415***	0.120
Number of participants	445			
Number of observations	10 680			
Log likelihood	− 2338.818			

*P < 0.10; **P < 0.05; ***P < 0.01

The standard deviations of monthly income, city location, having *bianzhi*, and having light working strength were statistically significant, indicated the existence of preference heterogeneity among the final year undergraduate nursing students.

### Estimated willingness to pay for job attributes

The results of WTP are shown in Table 5 and are used for relative comparisons. They were willing to pay RMB 2096.2 (US$ 310.1) monthly income for a job with light working strength than a job with heavy working strength. If the work environment improved from poor to excellent, they were willing to forego RMB 1572.4 (US$ 232.6) per month. In addition, they put a value of only RMB 702.4 (US$ 103.9) per month on a work location in the city, as compared to work in a township or village.

**Table 5:** Estimated willingness to pay (WTP) for job attributes among final year undergraduate nursing students (based on mixed logit estimates)

Attributes	WTP (RMB)	Lower level of 95% confidence interval	Upper level of 95% confidence interval
Location (ref: Township or village)			
County	605.0	362.1	845.6
City	702.4	433.9	968.5
*Bianzhi* (ref: No)			
Yes	1341.5	1118.6	1570.9
Career development and training opportunity (ref: Insufficient)			
Some	208.8	− 33.2	461.9
Sufficient	1350.0	1075.0	1626.6
Work environment (ref: Poor)			
Normal	1219.7	962.4	1475.6
Excellent	1572.4	1311.0	1847.5
Working strength (ref: Heavy)			
Medium	2085.8	1815.0	2365.8
Light	2096.2	1819.3	2 389.3

Note: According to the OECD data, the average annual exchange rate between US$ and RMB in 2017 was: US$ 1 = RMB 6.76

### Subgroup analysis

The results of mixed logit models and WTP among different subgroups are showed in Table 6. Apart from the attribute of location, the preferences for other job attributes between different subgroups were relatively similar. Students who came from the county or city valued a city location very highly with a willingness to pay of RMB 1594.2 (US$ 235.8) (95% CI, 1037.2–2 185.9), compared to students who came from townships or villages with a willingness to pay of only RMB 366.6 (US$ 54.2) (95% CI, 63.3–662.2). Compared to students whose family income was less than RMB 50 000 (US$ 7396), students with a family income of more than RMB 50 000 (US$ 7396) were willing to pay RMB 611.3 (US$ 90.4) (95% CI, 530.7–709.2) extra monthly income for a job in the city.

**Table 6:** Results of mixed logit models and willingness to pay (WTP) by different subgroups

Attribute	Hometown: township or village			Hometown: city or county			Family income (RMB per year): < 50 000			Family income (RMB per year): > 50 000		
	Mean	SD	WTP	Mean	SD	WTP	Mean	SD	WTP	Mean	SD	WTP
Monthly income	0.000489***	0.000236***	–	0.000469***	0.000261***	–	0.000460***	0.000231***	–	0.000554***	0.000271***	–
Location (ref: Township or village)												
County	0.217***	0.021	443.4	0.495***	0.207	1 055.5	0.219***	0.032	474.9	0.459***	0.131	829.7
City	0.179**	0.272	366.6	0.748***	0.754***	1 594.2	0.226***	0.508***	490.1	0.610***	0.496**	1 101.4
*Bianzhi* (ref: No)												
Yes	0.682***	0.554***	1 394.4	0.565***	0.758***	1 204.1	0.553***	0.575***	1 201.0	0.886***	0.733***	1 601.5
Career development and training opportunity (ref: Insufficient)												
Some	0.062	0.042	126.0	0.198*	0.031	421.5	0.126*	0.043	274.0	0.034	0.086	61.5
Sufficient	0.679***	0.038	1 389.5	0.599***	0.060	1 277.0	0.631***	0.211	1 371.6	0.693***	0.024	1 251.5
Work environment (ref: Poor)												
Normal	0.610***	0.024	1 248.9	0.557***	0.024	1 187.6	0.575***	0.015	1 250.1	0.686***	0.272	1 238.9
Excellent	0.725***	0.012	1 483.4	0.815***	0.065	1 737.0	0.770***	0.001	1 673.7	0.787***	0.271	1 421.2
Working strength (ref: Heavy)												
Medium	1.069***	0.016	2 187.6	0.900***	0.124	1919.2	1.015***	0.012	2 205.5	1.043***	0.002	1884.3
Light	0.977***	0.346**	1999.0	1.105***	0.524**	2 357.1	1.042***	0.417**	2 265.4	1.010***	0.392	1825.5
Participants	309			136			297			148		
Observations	7416			3264			7128			3552		
Log likelihood	− 1 581.716			− 739.695			− 1 590.238			− 741.323		

*P < 0.10; **P < 0.05; ***P < 0.01

Note: According to the OECD data, the average annual exchange rate between US$ and RMB in 2017 was US$ 1 = RMB 6.76

### Changes in uptake rates for a rural job versus a city job under different policy interventions

**Fig. 2:**
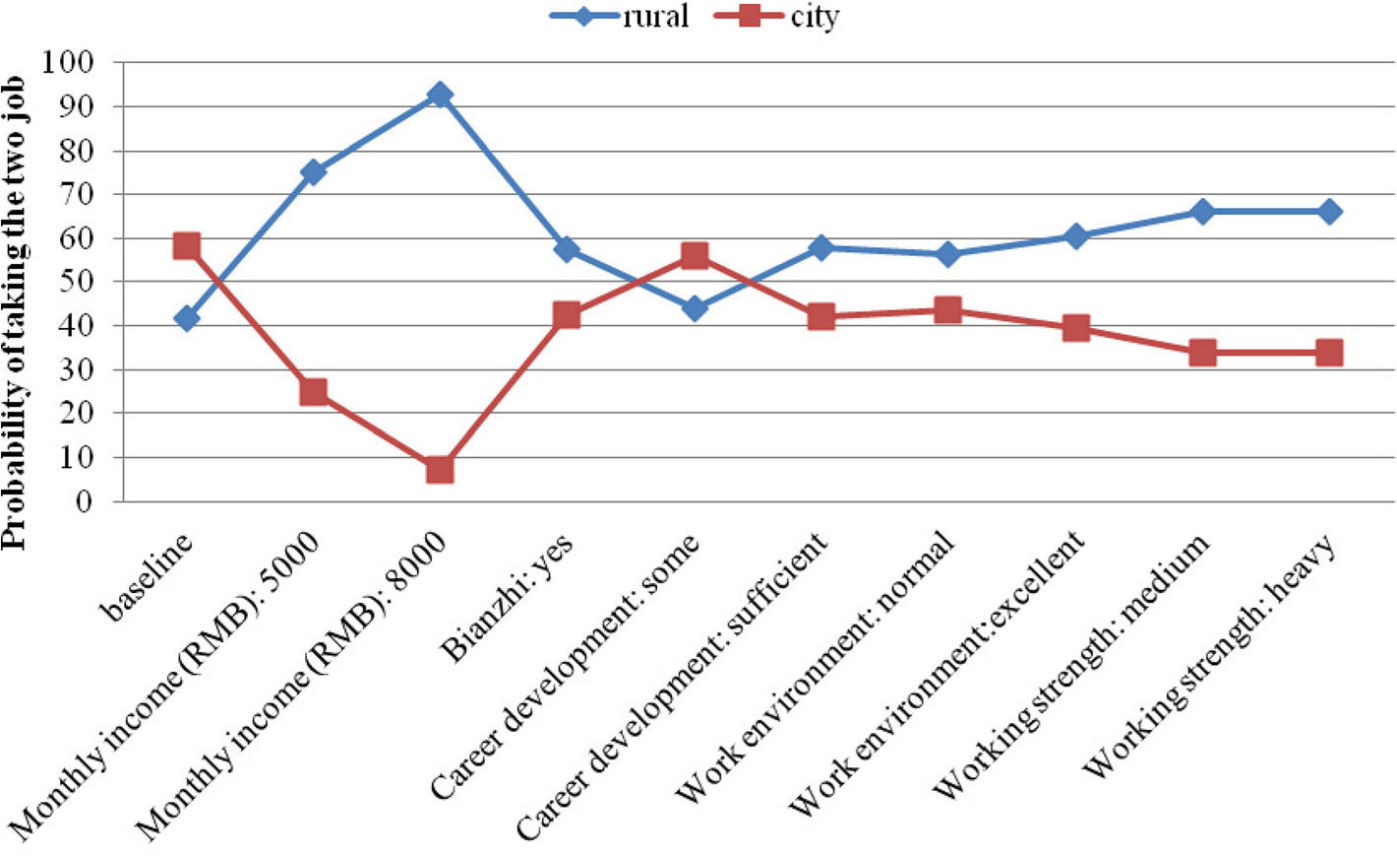
Figure that demonstrates the change in probabilities of taking the two job, as condition in rural job improve (In this baseline, both jobs have a monthly income of RMB 2000, no *bianzhi*, insufficient career development and training opportunity, poor work environment and heavy working strength)

Figure 2 shows the varying probabilities of taking a rural (township or village) job versus a city job, with various job conditions; the county job was omitted here but can be calculated based on the regression coefficients reported in the paper. It shows that the baseline probability of taking the rural job is 41.6%, and the probability of taking the city job is 58.4% (all else been equal). If monthly income increased from RMB 2000 (US$ 296) to RMB 5000 (US$ 740), the probability of choosing a rural job would increase to 75.2%. However, raising monthly income by another RMB 3000 (US$ 444) to RMB 8000 (US$ 1183), the probability would increase to 92.8%. The model predicted that a job with light working strength rather than heavy would increase the proportion of students opting a rural job to 66.2%. Improving the work environment from poor to excellent, career development and training opportunity from insufficient to sufficient and offering *bianzhi* would increase the probability of taking a rural job to 60.4%, 57.8%, and 57.7%, respectively.

## Discussion

This study has elicited preferences for job attributes among the final year undergraduate nursing students using a DCE. All six attributes (including both economic and non-economic factors) significantly affected the job choices of the students. They preferred a job with higher monthly income, light working strength, excellent work environment, sufficient opportunity for career development and training, *bianzhi*, and city location. The mixed logit model estimates further suggest the existence of preference heterogeneity in all six attributes. Comparing our results with what have been reported in the literature, all studies have included both monetary and non-monetary attributes in the nursing job choice DCEs; in addition, consistent findings suggest that monetary attribute (e.g., income) had significantly positive impact on the nursing job preference. On the other hand, the non-monetary attributes included in DCE studies varied in the literature (e.g., *bianzhi* attribute has been included in all studies focusing China, while housing attribute has been commonly included in studies focusing on African countries), partially highlighting the importance of taking into account of the country-specific context. For the same attribute been investigated in different DCEs (e.g., working strength), results are also mixed regarding to the significance and the relative importance.

Similar with other studies [28, 34], our findings confirmed that monthly income had a significant impact on the job choices of undergraduate nursing students. Wu et al. conducted a survey among 164 eligible nursing undergraduates who took internship in a tertiary hospital in Tangshan, and the monthly income was regarded as the most important attribute [28]. Apart from this, non-economic factors were also important. In particular, the work environment and working strength were the most important two non-economic job attributes for students. This result differs from what has been reported by Vujicica et al. [25], in which the authors found that the workload was valued the least important. One possible reason is that newly employed nurses suffer occupational stress from a heavy workload and they also more likely to work night shift in China [40]. It should also be noted that in the ranking exercise, these two attributes were not rated as more important than other attributes. This difference can be partially caused by the methodological differences between two approaches, in which respondents focused on one attribute at a time whereas in the DCE, respondents focused on whole scenarios which consisted of different attributes and more close to real world scenarios [41]. Further qualitative study is required to understand the difference in more detail.

According to the DCE result, policy interventions focusing on providing a better work environment will be an effective approach to attract nursing students. Previous studies conducted in Indonesia [34] and Uganda [22] found that nursing students preferred supportive management and an advanced quality facility over other non-economic attributes. In this study the attribute of work environment also included superior-subordinate relationship and colleagueship. A previous study has found that good relations with peers played a positive influence on recruiting and retaining registered nurses in rural areas [42]. The importance of healthy relationships at work is also supported by focus group discussions.

The *bianzhi* was found to have relatively minor effect, which seems contrary to other Chinese studies [26, 28]. This result may be partly explained by Maslow’s hierarchy of needs [43]. At present, the final year undergraduate nursing students are seeking a good job, and fundamental needs are not satisfied. Furthermore, limited students can find a job with *bianzhi* at present. Apart from this, all of the nursing students were born after 1990, and they have vitality and self-confidence. For this younger generation, a job with *bianzhi* may not be important for them. Therefore, economic incentives and providing a work with lighter working strength and better work conditions may more easily attract nursing students.

Interestingly, the preference of nursing students for location was the least important and was contrary to what was expected. The subgroup analysis suggested substantial heterogeneity among nursing students for the valuation of location, students who came from the city or county and with a family income of more than RMB 50 000 (US$ 7396) were willing to pay more monthly income for a job in city. Former DCE studies in Liberia and Vietnam also found that nurses and physicians born in rural areas were willing to pay less to work in urban areas [25, 44]. Nursing students had different family background and job expectation; students who lived in rural areas probably have planned to take up jobs close to their home. Furthermore, as the family income of most students was less than RMB 50 000 (US$ 7396), they urgently needed to find a steady job to earn more money. A follow-up in-depth qualitative study would be helpful to explain the reasons behind the observations.

It is crucial to recruit and retain health workers in rural areas [45]. Providing economic incentive to nursing students should be a priority of the Chinese government. This study found that raising monthly income from RMB 2000 (US$ 296) to RMB 5000 (US$ 740) would increase the probability of choosing a rural job to 75.2%. Unsatisfied remuneration among nurses in China could be the underlying reason that caused the above phenomenon [46, 47]. However, increasing monthly income alone may not be the most efficient way to attract more nursing students to rural areas. A similar study conducted in Tanzania also reached the result [35]. It may be wise for the government to focus on other policy interventions after raising the monthly income to a certain level. For example, recruiting nursing students from rural areas may be an effective way. Review papers for health workers’ attraction and retention also mentioned that rural upbringing increases chances of health workers returning to practice in rural areas [48, 49].

It is worth mentioning that any single intervention attracting health workers to take jobs in rural areas is unlikely to be sufficient or successful. Effective interventions need to combine different packages of policy interventions, and these interventions need to be matched with health workers’ preferences and expectations [45]. Different countries require completely different combinations of human resources policies so standard strategies which can be applied in any context are not realistic [21].

This study has three limitations. Firstly, it was conducted in medical colleges in Shandong Province which is located in eastern China; thus, these findings cannot be generalized to the whole country. Secondly, this study focused only on the job preferences of undergraduate nursing students. Their job preferences may differ from the current registered nurses. Thirdly, similar to other DCEs studies, we only have investigated the stated preferences of the students. Further research should compare these results with the revealed preferences based on actual behavior.

## Conclusion

This study empirically estimated job preferences of the final year undergraduate nursing students in China using a DCE and found that not only the economic factor but also non-economic factors significantly affected the job choices of nursing students. These results can provide useful information for policymakers to gain insight into their job preferences, and refine the employment policies. Apart from raising the monthly income to a certain level, prioritizing strategies which can reduce the working strength and improve the work environment may be more effective in China.

## Additional file

Additional file 1: **Table S1.** Table that demonstrates the results from a mixed logit model of discrete choice experiment data from all final year undergraduate nursing students. (DOCX 15 kb)

## Abbreviations

DCE: Discrete choice experiment
SD: Standard deviation
SE: Standard error
WTP: Willingness to pay
