# Supplementary material for: Job preferences of undergraduate nursing students in eastern China: a discrete choice experiment
Source: Hum Resour Health. 2019 Jan 3;17:1. doi: 10.1186/s12960-018-0335-3 (PMC6318922; doi:10.1186/s12960-018-0335-3)
Supplement: Supplementary file 1 — Table S1. Table that demonstrates the results from a mixed logit model of discrete choice experiment data from all final year undergraduate nursing students. (DOCX 15 kb) [file 12960_2018_335_MOESM1_ESM.docx]

**Table S1** Results from a mixed logit model of discrete choice experiment data from all final year undergraduate nursing students

| **Attributes** | **Mean coefficient** | **Standard error** | **Standard deviation** | **Standard error** |
| --- | --- | --- | --- | --- |
| **Monthly income** | 0.000456^***^ | 0.000020 | 0.000234^***^ | 0.000018 |
| **Location (ref: Township or village)** | | | | |
| County | 0.250^***^ | 0.055 | 0.002 | 0.183 |
| City | 0.358^***^ | 0.060 | 0.473^***^ | 0.100 |
| ***Bianzhi* (ref: No)** |  |  |  |  |
| Yes | 0.587^***^ | 0.052 | 0.574^***^ | 0.065 |
| **Career development and training opportunity (ref: Insufficient)** |  |  |  |  |
| Some | 0.111^**^ | 0.054 | 0.060 | 0.194 |
| Sufficient | 0.677^***^ | 0.063 | 0.127 | 0.293 |
| **Work environment (ref: Poor)** |  |  |  |  |
| Normal | 0.583^***^ | 0.058 | 0.001 | 0.143 |
| Excellent | 0.778^***^ | 0.059 | 0.014 | 0.156 |
| **Working strength (ref: Heavy)** |  |  |  |  |
| Medium | 0.888^***^ | 0.060 | 0.004 | 0.144 |
| Light | 0.907^***^ | 0.065 | 0.366^***^ | 0.121 |
| **Number of participates** | 507 | | | |
| **Number of observations** | 12168 | | | |
| **Log likelihood** | -2725.274 | | | |

^*^P<0.10; ^**^P<0.05; ^***^P<0.01
